# Experimental encephalomyelitis at age 90, still relevant and elucidating how viruses trigger disease

**DOI:** 10.1084/jem.20221322

**Published:** 2023-01-18

**Authors:** Lawrence Steinman, Roberto Patarca, William Haseltine

**Affiliations:** 1 Department of Neurology and Neurological Sciences and Pediatrics, Stanford University, Stanford, CA, USA; 2 Access Health International, Ridgefield, CT, USA

## Abstract

20 yr ago, a tribute appeared in this journal on the 70th anniversary of an animal model of disseminated encephalomyelitis, abbreviated EAE for experimental autoimmune encephalomyelitis. “Observations on Attempts to Produce Disseminated Encephalomyelitis in Monkeys” appeared in the *Journal of Experimental Medicine* on February 21, 1933. Rivers and colleagues were trying to understand what caused neurological reactions to viral infections like smallpox, vaccinia, and measles, and what triggered rare instances of encephalomyelitis to smallpox vaccines. The animal model known as EAE continues to display its remarkable utility. Recent research, since the 70th-anniversary tribute, helps explain how Epstein–Barr virus triggers multiple sclerosis via molecular mimicry to a protein known as GlialCAM. Proteins with multiple domains similar to GlialCAM, tenascin, neuregulin, contactin, and protease kinase C inhibitors are present in the poxvirus family. These observations take us a full circle back to Rivers’ first paper on EAE, 90 yr ago.

## Teaser

The animal model for experimental encephalomyelitis, first published 90 yr ago, continues to illuminate mechanisms of disease. The model, initially developed to understand how smallpox triggers neuroinflammation on rare occasions, now moves full circle to its origins.

Experimental autoimmune encephalomyelitis (EAE) has been catalytic for the development of therapeutic breakthroughs for multiple sclerosis (MS), including the first synthetic peptide–based polymer for MS, the first monoclonal antibody therapy for MS, and the first oral therapy for MS. EAE has also illuminated important mechanisms for immune tolerance, and the model has provided seminal observations on the impact of the microbiome on autoimmune disease. Before describing those important breakthroughs in therapeutics and the insights into the basic mechanisms underlying autoimmunity, where the EAE model provided the stage for the initial discoveries, we shall describe the various models themselves, collectively termed EAE.

## The two dominant models of EAE, active and passive

There are two dominant models in EAE, referred to as “active EAE” and “passive EAE.” The terms are unfortunate, but they have persisted. There is nothing passive about transferring immune cells which then paralyze an experimental animal. Neither active EAE nor passive EAE is a preferable model, and the term “passive” seems poorly chosen, in our opinion. Both models are often used to answer an experimental question with two independent systems. The drawback of the active model is that it requires an adjuvant to sensitize animals reliably and easily after a single injection. 90 yr ago, Rivers’ initial efforts were in non-human primates without the use of an adjuvant and required multiple injections (Rivers et al., 1933; Steinman, 2003). EAE has been conducted mostly in rodents for the past 50 yr, particularly in the mouse, where different genetic strains are most extensive. The passive model entails injection of T cells raised in an animal initially injected anyway with an adjuvant and a myelin protein or peptide. In the passive model, these myelin-specific T cells are often clones, often expanded in vitro after removal from a donor that had been injected with antigen in adjuvant. The transferred cells are highly specific for a myelin antigen. However, the “naive” recipient in the passive model has not been injected with adjuvant and thus does not have the systemic perturbation of the immune system, which develops after the use of a powerful adjuvant.

Thus, Rivers repeatedly injected brain tissue and brain extracts in his initial description of the model (Rivers et al., 1933). The experimental disease was called acute disseminated encephalomyelitis, a term still in use in neurology practice, as this disease is seen in humans. When Kabat and colleagues first published on the use of Freund’s adjuvant in monkeys in the EAE model, the scientific community still referred to the model as experimental “disseminated encephalomyelitis” (Kabat et al., 1947). The term “experimental allergic encephalomyelitis” was first seen in 1947 and 1948 (Morgan 1947; Jervis and Koprowski, 1948). The first use of the term “autoimmune encephalomyelitis” appeared in French in 1978 (Staykova et al., 1978). Over the next decade, scientists gradually used EAE to mean “experimental autoimmune encephalomyelitis.” Thus, when EAE is induced with a preparation of a myelin antigen in Freund’s adjuvant, it is called active EAE, where the “A” now stands for autoimmune. Subsequently, a passive model of EAE was developed whereby lymphocytes taken from animals sensitized to myelin and then transferred to naive recipients would develop paralysis (Levine et al., 1968). The transferred lymphocytes come from an animal that has received myelin antigens in Freund’s adjuvant.

Passive EAE models have evolved, and now it is common to induce EAE with cloned T cells or with experimental animals where the TCR genes have been installed as transgenes. Over 40 yr ago, the first demonstration of clonable T cells to myelin proteins was introduced, with the first example being the seminal publication in 1981 by Ben-Nun, Cohen, and Wekerle (Ben-Nun et al., 1981). This was followed by several papers where actual T cell clones were used to induce EAE (Zamvil et al., 1985). Then their TCRs and homing molecules were elucidated. The TCRs that recognize myelin proteins were sequenced, and transgenic mice expressing these receptors were constructed. The transgenic mice developed spontaneous EAE (Goverman et al., 1993).

## Deployment of the two models of EAE to develop therapeutics for MS

The passive EAE model played a key role in the development of the first monoclonal antibody to treat MS. Initially, passive EAE was induced with clones of T cells reactive to myelin. Attempts to block the entry of these T cells from blood to the central nervous system (CNS) were analyzed with binding assays on frozen sections of material from EAE brain, and the α4 integrin molecule was determined to be critical. Natalizumab was approved in 2004 for the treatment of MS with monthly intravenous infusions (Yednock et al., 1992; Steinman, 2012).

Active EAE, on the other hand, was instrumental in the development of the first synthetic copolymer for treatment of relapsing MS (Teitelbaum et al., 1971), and the first orally available pharmaceutical, fingolimod, for treatment of relapsing MS. Sela, Arnon, and colleagues developed a synthetic copolymer that suppressed active EAE when given intravenously. The copolymer, named glatiramer, was approved in 1996 after 25 yr of development. Studies in active EAE opened the path for the first oral disease-modifying drug in MS (Teitelbaum et al., 1997). Promising results in experiments employing active EAE (Fujino et al., 2003) allowed for a change in direction of pharmaceutical development of a sphingosine phosphate modulator, fingolimod, that was to become the first orally available approved drug for MS. Initial studies in transplant rejection were unimpressive. However, based on decisive experiments in the active EAE model, where paralysis was essentially totally ameliorated in rodents, a development program for this compound in MS ensued at Novartis. The program succeeded in the clinic and led to regulatory approval worldwide of a once-daily oral drug for MS (Chun and Brinkman, 2011).

Thus, the first monoclonal antibody treatment for relapsing MS, approved in 2004, came from the passive EAE model. The first synthetic peptide–based drug for MS was approved in 1996, initiated in work on the active model of EAE in 1971 (Teitelbaum et al., 1971). The first oral drug for MS, approved in 2010, also emerged from the active EAE model. These achievements came from pivotal studies in the two major forms of EAE, active and passive. Together, these three landmark approved treatments have been used by over a million individuals with MS. That is an astonishing tribute to this 90-yr-old model, in our opinion.

## The role of EAE in elucidating autoimmune mechanisms

Once the TCRs that recognize myelin proteins were sequenced, transgenic mice expressing these receptors were constructed that developed spontaneous EAE (Goverman et al., 1993). These TCR transgenic mice, where the TCRs for myelin-specific T cells were engineered into wild-type mice, were critical in helping to understand the gut microbiota. Spontaneous EAE in these transgenic mice was observed, but only when the T cells were activated by the commensal microbiome in the gut. Germ-free mice never developed paralysis in the EAE model. This was one of the compelling demonstrations of the effect of the microbiome in triggering autoimmune disease, in this case in the CNS (Berer et al., 2011). Understanding how various microbiomes might influence autoimmune disease is one of the fruits harvested from some of the passive models of EAE using cloned myelin-specific T cells and then mice that are transgenic for the TCRs associated with T cells that can paralyze an experimental animal. One can contend that EAE provided the first exciting proof of the dramatic influence of the microbiome on autoimmunity.

The concept of “epitope spreading,” also referred to as “determinant spreading,” was another gift in which the EAE model played a starring role. 30 yr ago, Sercarz and colleagues (Lehmann et al., 1992) showed that in a relapsing model of active EAE, after immunization with a peptide from myelin, the first immune attack is focused on that peptide. But as disease progresses and becomes chronic, immunity arises without further immunization to other regions of the same myelin protein and then to other myelin proteins. This is termed determinant spreading. A later study by Tuohy and colleagues (Tuohy et al., 1999; Steinman, 1999) concluded that chronic progression of EAE and MS involves a shifting of autoreactivity from a primary initiating determinant on a self-molecule to new regions of the same molecule and other molecules, not only on myelin but on neurons. This process might be responsible for disease progression and for worsening of disability over time.

30 yr ago, as noted above, the passive EAE model illuminated the critical role of the microbiome in determining whether clinical disease might be manifest or remain quiescent even after T cells capable of attacking myelin were present in an experimental animal (Berer et al., 2011). More recently, in 2022, EAE has helped illuminate how a virus triggers the human disease, MS.

Returning to the theme that inspired Rivers and colleagues 90 yr ago, EAE has provided an important animal model for helping to elucidate how the EBV triggers the most prevalent neuroinflammatory disease MS (Bjornevik et al., 2022; Lanz et al., 2022; Robinson and Steinman 2022). The first positive result linking EBV to MS was published in 1980 by researchers at University of California, Los Angeles, who showed that there were increased antibodies to EBV in MS patients compared with controls (Sumaya et al., 1980). Recent work published in the winter of 2022 showed that EBV is the trigger for MS (Bjornevik et al., 2022; Robinson and Steinman, 2022). In a study of over 10,000,000 individuals from the U.S. military, 800 of 801 individuals who developed MS were infected with EBV. Moreover, 35 individuals who entered the military EBV negative on serological testing then went on to develop MS. 34 of those 35 individuals turned EBV positive on serology at some point before a diagnosis of MS was made.

Epstein Barr Nuclear Antigen-1 (EBNA-1) has a region of molecular similarity with an antigen in the white matter of individuals with MS, called GlialCAM. Investigators (Lanz et al., 2022) built their research on yet another observation first published in *JEM* in 1930 by Jules Freund, working at the University of Pennsylvania. Freund called attention to the presence of antibody in the CNS when he demonstrated that he could isolate antibodies from the brain and spinal cord of rabbits, who were immunized against typhoid (Freund, 1930). Over the next quarter of a century, accumulation of clonal antibodies was described in the spinal fluid of individuals with MS and in a few other rare diseases. Lanz et al. (2022) analyzed the heavy and light chains of these clonal antibodies and then tested these antibodies to see what the antibodies could bind. Major clonal antibodies were isolated and sequenced from the cerebrospinal fluid. 37 of the 148 clonal antibodies that were isolated from cerebrospinal fluid recognized EBNA-1. Many of these monoclonal antibodies in the cerebrospinal fluid that bound EBNA-1 also bound to GlialCAM, which has a peptide sequence that mimics a region in EBNA-1 (Lanz et al., 2022). The remarkable similarity in structure between a region of a virus and a region on a self-protein is termed molecular mimicry. Molecular mimicry is one of the pervasive ideas on how the immune system mistakes self- from non-self-antigens (Steinman, 1993).

To help confirm the role of GlialCAM, the investigators took a lesson from Robert Koch. “Koch’s postulates” in their purest application would require that the disease-causing microbe with some of its products be introduced into a suitable host to reproduce the disease. Thomas Rivers, the discoverer of EAE, addressed Koch’s postulates (Rivers, 1937) in a speech to the American Society of Bacteriologists.

Though it is not possible to infect a normal experimental rodent with EBV, we nonetheless were able to demonstrate that injection of the EBNA-1 peptide that mimicked a myelin-associated protein, GlialCAM, would worsen paralysis in an active model of EAE. The active EAE model was useful for helping to emphasize the potential role of a region of EBNA-1 that is a molecular mimic of GlialCAM. Administration of the peptide comprising the molecular mimic worsened paralysis in the active EAE model. Koch’s postulates (Koch, 1884) and Rivers’ exposition of this idea (Rivers, 1937) inspired this experiment. Not all of Koch’s postulates were fulfilled, and those experiments were not attempted. However, translating the human studies to the EAE model was a cornerstone in the demonstration that this region of EBNA-1 could worsen clinical neuroinflammation. Such an experiment is actually a reverse translation of intriguing human data back to the classic model of neuroinflammation in an experimental animal.

## Future therapeutics: Promising results in EAE with nucleic acid “tolerizing vaccines”

Subtle changes in how a protein is exposed to the immune system can be critical in inducing or protecting against a disease. This has been tested in both MS and EAE. Extensive studies on triggering EAE and on reversing paralysis in EAE with alterations of a myelin peptide have been undertaken. Altered peptides of molecular mimics between myelin and various viruses were able to suppress both passive EAE and active EAE (Karin et al., 1994; Brocke et al., 1996; Ruiz et al., 1999). These experiments have been translated from the EAE model to humans with MS a quarter of a century ago.

However, when an altered peptide of myelin basic protein was tested in two trials in relapsing MS, disturbing results were observed. In one trial, when the altered myelin peptide was given at 5 mg weekly via subcutaneous injection, there was evidence of reduced inflammatory lesion activity on magnetic resonance imaging (MRI), a long-sought-after result and the primary endpoint in the study. However, the trial was discontinued due to hypersensitivity reactions in 13 of the 142 subjects (9%; Kappos et al., 2000), despite the promising effects on neuroinflammation. In a trial with the same altered peptide given at 50 mg weekly subcutaneously, three patients out of seven developed transient clinical worsening of their relapsing MS, with dramatic increases in inflammatory lesions on MRI (Bielekova et al., 2000). The worsening resolved, but the discordance between what was seen in EAE in the preclinical work and what was observed in the clinic serves as a reminder that not all promising EAE experiments translate to success when tested in the clinic.

Further work in the EAE model showed how hypersensitivity reactions could culminate in a new version of “horror autotoxicus,” first described by Ehrlich (Himmelweit, 1960). Anaphylaxis elicited with self-peptides from myelin and other proteins helped to understand this phenomenon. These experiments with EAE demonstrated that in driving a T helper 2 type response to self, this strategy may terminate in anaphylaxis (Pedotti et al., 2001; Pedotti et al., 2003). Mechanistic studies showed that self-peptides that drive anaphylaxis were not presented in the thymus and thus escaped what is called “central tolerance,” which occurs in the thymus (Pedotti et al., 2001; Pedotti et al., 2003).

After the alarming results with administration of actual peptide domains of myelin antigens to individuals with MS (Bielekova et al., 2000; Kappos et al., 2000), a new approach was developed using nucleic acids to encode the myelin proteins. EAE was the initial testing grounds for these novel approaches with DNA and RNA constructs to tolerize the immune system.

Administration of myelin antigens with modified nucleic acids has been successful in suppressing EAE and has even been taken into the clinic in relapsing MS. A modified DNA plasmid was engineered in these experiments in mice and then in human trials. In the coding region of the DNA plasmid were sequences for the four major myelin proteins. We engineered the non-coding region to have fewer immunostimulatory DNA motifs. Thus, we reduced the number of inflammatory CpG hexanucleotide sequences. These sequences stimulate the innate immune system by binding to TLR9 (Garren et al., 2008). The experiment was successful in active EAE models. In the preclinical models, immune responses to myelin antigens were reduced and clinical paralysis was ameliorated (Robinson et al., 2003). In an editorial about the paper by Robinson and colleagues (Robinson et al., 2003) on DNA vaccines for tolerization, Sercarz (2003) described both intra- and intermolecular spreading to other myelin antigenic determinants “in the neighborhood,” referring to other proteins in the myelin sheath. When the engineered DNA plasmid was given, Robinson and colleagues had shown that the tolerizing DNA induced “epitope (determinant) contraction.” This means that there was a reduction in the amount of antibodies to various myelin proteins (Robinson et al., 2003), likely accounting for the reduction in clinical paralysis.

These results on tolerization to myelin proteins in EAE were advanced to the clinic. In a phase 2 trial on 267 patients with a DNA vaccine designed to tolerize to myelin basic protein, the investigators reported some intriguing evidence of “inverse vaccination” with reduced inflammatory activity seen on MRI and with reduction of anti-myelin antibody in the cerebrospinal fluid (Garren et al., 2008), similar to what was observed in the preclinical experiment (Robinson et al., 2003).

More recently, mRNA constructs have been engineered to induce tolerance. Nanoparticle-formulated 1 methylpseudouridine–modified mRNA coding for disease-related myelin autoantigens suppressed both active and passive EAE (Kreinke et al., 2021). Thus, by careful engineering of a nucleic acid formulation, it is possible to tolerize the immune system to unwanted autoimmune responses. The EAE model has again led the way.

Thus, scientists working with the EAE model are already transporting us into a future in which we might have therapeutic inverse vaccines to induce antigen-specific tolerance with nucleic acid constructs. One can imagine a race to develop DNA and RNA tolerizing vaccines. Individuals with MS and other autoimmune diseases may benefit from this “competitive race” between DNA and mRNA tolerizing vaccines. Instead of an “arms race,” we might have for the benefit of mankind a “race to tolerize,” with immunology taking center stage, as it has during the recent pandemic.

## Poxviruses and their ability to infect oligodendroglia and to impact myelin physiology

In line with what Rivers and colleagues began in their 1933 paper (Rivers et al., 1933), EAE models can be used to study poxviruses and their effects on oligodendroglia and myelin physiology. Based on bioinformatic analyses, poxviruses might contribute to demyelination by encoding proteins similar to cellular ones that have been associated with MS pathogenesis in EAE models and humans (summarized in the box).

**Neuropathogenic mechanisms of poxviruses**

(1) Tenascin-like inhibitors of myelination
(2) Neuregulin-like inhibitors of monocyte migration
(3) Contactin-1–like paranodal proteins at the node of Ranvier in myelinated axons and Serpin-like response modifiers
(4) Protein C inhibitors driving neuroinflammation

**Figure 1.**
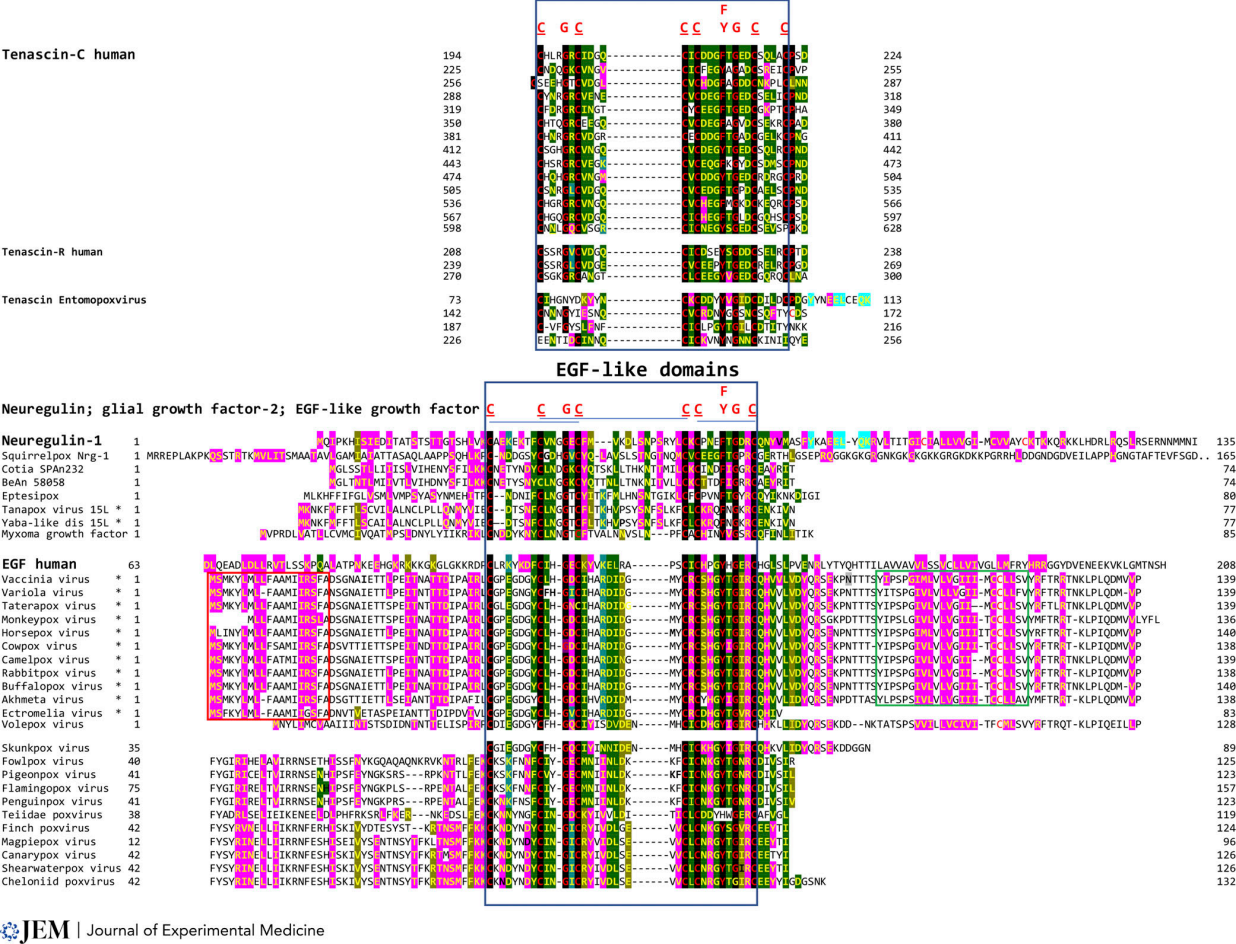
**Presence of a poxviral gene encoding a tenascin-like protein in an entomopoxvirus and of genes encoding neuregulin-like proteins and other EGF-like domain-containing growth factors in poxviruses infecting humans and other animals.** A hallmark of EGF domains is the presence of six cysteine residues. Conserved amino acids in EGF-like domains in human and poxviral tenascins, neuregulins, and growth factors are shown in red letters at the top of sequence alignments and tallow letters highlighted in black. Other conserved positions among the proteins shown are highlighted in dark or light green or pink. The blue box indicates EGF-like domain in all proteins shown, while the red box is leader sequence, and the green box is transmembrane segment present in mostly poxviruses infecting humans. Asterisks indicate poxviruses known to infect humans or human cells. Annotations, amino acid lengths, and accession numbers for the proteins shown are shown in Table S1.

First, myelination requires migration, proliferation, and differentiation of oligodendrocyte precursor cells and is influenced by glycoproteins and proteoglycans of the extracellular matrix. In particular, the extracellular matrix glycoprotein tenascin-C has an inhibitory effect on the differentiation of oligodendrocyte precursors and the remyelination efficiency of oligodendrocytes (Bauch and Faissner, 2022). The alphaentomopoxvirus isolated from the beetle *Anomala cuprea* encodes a tenascin-like protein (Mitsuhashi et al., 2014; Fig. 1).

The tenascins contain repeats of epidermal growth factor (EGF)–like domains, which are commonly found in the extracellular milieu, where they participate in signaling pathways through protein–protein interactions; comprise 30–40 amino acids; include six cysteine residues that typically form three disulfide bonds; and have as main secondary structure a two-stranded β-sheet followed by a shorter one (Tombling et al., 2019). There are 14 repeats of EGF-like domains in human tenascin-C, three in tenascin-R (whose receptor is contactin-1; Zacharias and Rauch, 2006), and four in the entomopoxviral tenascin-like protein (Fig. 1). Tenascin-C upregulates expression of matrix metalloproteinase that cleaves tenascin-C into fragments containing the EGF-like domains with proapoptotic activity (Wallner et al., 2004). Matrix metalloproteases play a key role in the penetration of immune cells into the brain in MS and EAE. Inhibition of matrix metalloproteases suppresses EAE (Gijbels et al., 1994).

Although the tenascin-like gene has been documented thus far only in an insect poxvirus, its jump from this or poxviruses from other hosts to poxviruses that infect humans might be plausible because insects can function as vectors of poxviruses among mammals (Fischer et al., 2021; Oliveira et al., 2017). There is also great plasticity in gene transfer involving poxviruses, as illustrated by gene transfer between poxviruses and baculoviruses in insects (Thézé et al., 2015), and poxvirus-mediated horizontal transfer of retroposons from reptiles to mammals, including humans (Piskurek and Okada., 2007).

Second, several poxviruses encode human neureugulin-1 (NRG-1)–like proteins (Fig. 1), which also contain an EGF-like domain, with a different distribution for the six conserved cysteine residues and their disulfide bonds through which they could bind to ErbB3 and ErbB4 receptors (Burden and Yarden, 1997). The poxviral proteins closest to NRG-1 in the EGF-like domain (Fig. 1) might compete with NRG-1 for binding to ErbB receptors, thereby adversely affecting myelination. NRG-1 regulates the thickness of the myelin sheath (reviewed in Mei and Xiong, 2008). NRG-1 also has emerged as a new immune modulator in CNS injury. Kataria et al. (2021) and Viehover et al. (2001) demonstrated that levels of Nrg-1β1, a major isoform of NRG1 in the CNS, are significantly reduced in association with progression to relapsing–remitting MS in humans, as well as in EAE mice within spinal cord lesions, and peripherally in the plasma and spleen as disease progresses from presymptomatic to active phases of the disease. Systemic restoration of NRG-1β1 was sufficient to delay paralysis and reduce severity in EAE models. A non-inflammatory phenotype was observed in macrophages, T helper type 1 cells, and microglia in spinal cord lesions of active EAE. A large set of poxviruses, including those infecting humans (Lima et al., 2019; Oliveira et al., 2017), encode secreted proteins about the same size as human NRG-1 and with EGF-like domains more similar to that in EGF, and thus these proteins are most likely to bind to ErbB1 (Fig. 1; Zhang et al., 2017; Jeng et al., 2013; Haltom and Jafar-Nejad, 2015).

**Figure 2.**
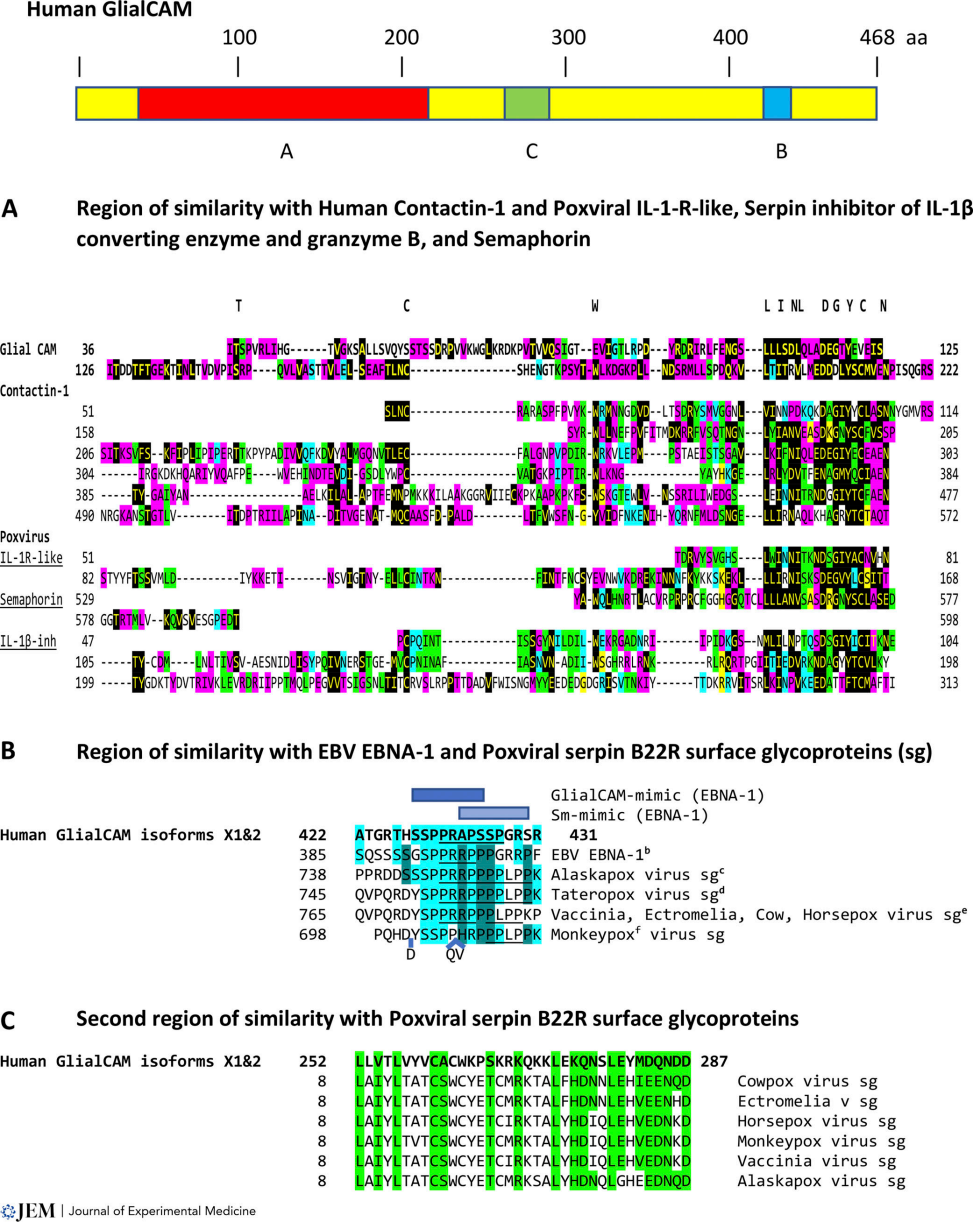
**Regions of similarity among human GlialCAM, EBNA-1, and poxvirus serpin B22R surface glycoproteins (sg), and among human GlialCAM, human contactin-1, and poxvirus IL-1R–like, serpin inhibitor of proteinases (IL-1β converting enzyme and granzyme B), and semaphorin.** Top: Schematic of human hepatocyte/GlialCAM (isoforms ×1 and 2) protein with regions of similarity in A–C. **(A)** Region of similarity among human GlialCAM, human contactin-1 (1007 aa; KAI065384), and poxviral IL-1R–like, serpin inhibitor of IL-1β converting enzyme and granzyme B, and semaphorin proteins. Conserved amino acid sequences among all or most of these proteins are depicted at the top of the panel and are shown with letters in yellow highlighted in black. Other identical or conserved amino acid positions between proteins are highlighted in pink, green, blue, or yellow. IL-1R–like proteins include those from lumpy skin disease virus (AAK43553 shown in figure; NW-LW: AAN02579; AAN02737; NI-2490: NP_150447; QNN94323; AYV61145) and Sheeppox Q2/1l (AAC32892). The semaphorin gene is from the eastern grey kangaroo poxvirus (YP_010085293). The serpin inhibitor of IL-1β converting enzyme and granzyme B is from cowpox (shown in figure: ADZ30596). **(B)** Region of similarity among human GlialCAM, EBV EBNA-1, and poxviral serpin B22R surface glycoproteins from poxviruses infecting humans. Identical amino acids or conserved amino acid substitutions among all sequences are highlighted in light blue, while those identical or conserved only between EBNA-1 and poxviral serpin B22R surface glycoproteins are highlighted in teal. Blue boxes indicate GlialCAM mimic region (dark) and Sm-mimic region (light). PXXP motifs are underlined. ^a^Human hepatocyte and glial CAM (XP_016872850); ^b^EBNA-1 (QAP52584.1); ^c^Alaskapox virus (recently isolated from a man in Alaska) surface protein (QED21095); ^d^Tateropox virus (surface protein: YP_717526) is closest to smallpox virus; ^e^Poxvirus membrane/surface/CPVX219/BR22R: Numbering corresponds to cowpox virus (CAB5514207), Ectromelia virus (mouse; QSV39764), Horsepox (ABH08313), and Vaccinia (DAD53327), which is closest to Horsepox; and ^f^Monkeypox MPXVgp182 (QNP13230). **(C)** The second region of similarity between human GlialCAM and serpin B22R surface glycoproteins from poxviruses infecting humans. Identical amino acids or conserved amino acid substitutions among sequences are highlighted in green. Accession numbers are as above.

Third, poxviruses encode factors that contribute to immune escape and determine host range while also potentially contributing to virus-induced demyelination (Oliveira et al., 2017). For example, human GlialCAM has a 186-amino-acid-long region of similarity to human contactin-1 as well as to IL-1R–like proteins (lumpy skin disease poxvirus); cowpox virus–encoded serpin cytokine response modifier [crm]A, which inhibits the endogenous IL-1β converting enzyme and the exogenous granzyme B proteinase delivered by cytotoxic T cells (Quan et al., 1995); and an eastern grey kangaroo poxvirus-encoded protein similar to semaphorin (Bennett et al., 2017; Fig. 2 A). Antibodies directed to semaphorins can block EAE (Okuno et al., 2010). Contactins and other proteins at the node of Ranvier are targeted in MS and Guillain–Barré syndrome (Steinman, 2009; Devaux et al., 2016; Manso et al., 2019).

Contactins are neural cell-adhesion molecules of the Ig superfamily, which as semaphorins (Iragavarapu-Charyulu et al., 2020) are regulatory molecules in the development of the nervous system and in axonal guidance and are also expressed by the immune and other systems. Contactins exist in membrane-bound and soluble forms, and their extracellular segment contains six Ig-like repeats similar to the Ig constant domains (Mercati et al., 2013), which is the segment that is similar to human GlialCAM and poxviral proteins affecting IL-1. Contactin gene expression is downregulated during myelination in vivo and in vitro (Einheber et al., 1997), and it is, therefore, possible that the poxviral proteins whose main task appears to be immune evasion for viral virulence could increase concentrations of contactin-like molecules, thereby negatively affecting myelination. Lower baseline serum contactin-1 concentrations have been associated with long-term disability progression during natalizumab treatment of relapsing–remitting MS (van Lierop et al., 2022), and patients with relapsing–remitting MS have significantly lower levels of semaphorin 3a compared with controls (Rezaeepoor et al., 2017). The poxviral IL-1R–like protein and inhibitor of IL-1β converting enzyme may, on the other hand, reduce the IL1β-mediated synaptic dysfunction, possibly leading to exitotoxic damage in both EAE and MS as well as neuroinflammation overall (De Vito et al., 2022; Mandolesi et al., 2017). In IL-1Ra^−/−^ mice with active EAE, IFN-γ, IL-17, and TNF-α production and proliferation were enhanced in IL-1Ra^−/−^ T cells (Matsuki et al., 2006).

Fourth, poxviruses including those infecting humans encode protein C inhibitor–like proteins. Proteomic analyses had identified protein C inhibitor within MS chronic active plaques, and in vivo administration of recombinant activated protein C reduced disease severity in an active EAE model (Han et al., 2008). Interactions between poxviruses, the coagulation cascade, and neuroinflammation constitute a frontier that beckons for further investigation.

## Poxviruses contain a similar molecular mimic associated with GlialCAM

In a remarkable confluence of observations, we return to the comment of Thomas Rivers that neurological symptoms and signs occasionally develop after smallpox, vaccinia, and measles. Moreover, according to the CDC (https://www.cdc.gov/smallpox/vaccine-basics/vaccination-effects.html), between 14 and 52 individuals out of a million developed serious reactions after the first smallpox vaccination. One of the rare serious reactions was encephalomyelitis. Only a fraction of the 14 to 52 individuals in a million who had a serious reaction developed encephalomyelitis. However, these rare reactions including encephalomyelitis were among the reasons that Rivers embarked on establishing the EAE model.

Of interest, a fragment similar to the 13–amino acid autoantigenic sequence shared between EBNA-1 and GlialCAM is present in the serpin B22R surface glycoproteins of poxviruses. This sequence with similarity to GlialCAM has been isolated from poxviruses in humans (Alaskapox and Taterapox viruses) and other species (cow, horse, mouse, and monkey) that infect humans (Fig. 2 B). Whether this poxviral fragment similarly functions as an autoantigen remains to be determined.

Poxviral serpins function as host range factors, are homologous to serine protease inhibitors, and have antiapoptotic and anti-inflammatory activities. Inactivation of B22R or B13R serpins in vaccinia virus reduces its virulence and extends host survival while not affecting immunogenicity (Legrand et al., 2004). The region that is similar among EBNA-1, human GlialCAM, and poxviral B22R serpins is proline-rich and includes the PXXP motif (P for Proline and X for any amino acid; underlined in Fig. 2 B), which is typically involved in protein–protein interactions, which might affect signaling pathways with potentially untoward consequences on myelination. Based on the unique features of proline and proline-rich segments, viral proteins with PXXP motifs have been proposed to play a role in MS (Pi et al., 2022). Poxviral B22R serpins (except for that from monkeypox, which only has one core PXXP) have the RXXPXXP motif, and GlialCAM has the PXXPXR motif; the charged arginine (R) side chain increases the binding affinity of the core PXXP motif.

GlialCAM and the serpin glycoproteins of poxviruses also have another 36-amino-acid-long segment of similarity between them (Fig. 2 C), which might constitute another autoantigenic region rich in amino acids with charged side chains and contribute to determinant spreading. The CXXXC and CXXC motifs (C for cysteine) in this second region have been associated in other viruses with protein interactions and thiol–disulfide transfer (Sobhy, 2016).

Poxviruses, whose infection prevalence in humans has been increasing since the World Health Organization recommended discontinuation of smallpox vaccination in 1980 (Shchelkunov, 2013; Diaz, 2021), may thus contribute to the pathogenesis of disseminated encephalomyelitis.

Remarkably, the EAE model, now in its 90th year, continues to provide unexpected insights into understanding human disease mechanisms, particularly in relation to inflammatory diseases of the central and peripheral nervous systems including MS and Guillain Barré Syndrome. What started as a model to help understand adverse reactions to smallpox vaccine may now be teaching us how poxviruses might actually trigger neurologic sequela.

We now might understand how disseminated encephalomyelitis rarely followed smallpox infection, and how it was triggered, albeit rarely, by both smallpox and smallpox vaccination (Booss and Davis, 2003). Immunity to the GlialCAM module in smallpox might be a key trigger for encephalomyelitis. Most recently, there has been a report of two individuals who developed disseminated encephalomyelitis after monkeypox viral infection (Pastula et al., 2022). As noted, the monkeypox virus contains the GlialCAM-like PXXP module.

## Coda

The EAE model continues to help explain pathogenic mechanisms. It has given us remarkable insights into the microbiome and autoimmunity. It has provided the field of immunology with the concept of determinant spreading. It has provided vivid examples of molecular mimicry as triggers for autoimmunity after infection. It has provided a new version of horror autotoxicus. The first synthetic polymeric peptide for treatment of MS came from the model, as well as the first monoclonal antibody to treat MS and the first oral drug for MS (Fig. 2). These three therapeutics for MS have been taken by over a million individuals with MS. EAE has served as a proving ground to test modern DNA and RNA tolerizing vaccines, which may point to another significant chapter in the distinguished history of EAE.

**Figure 3.**
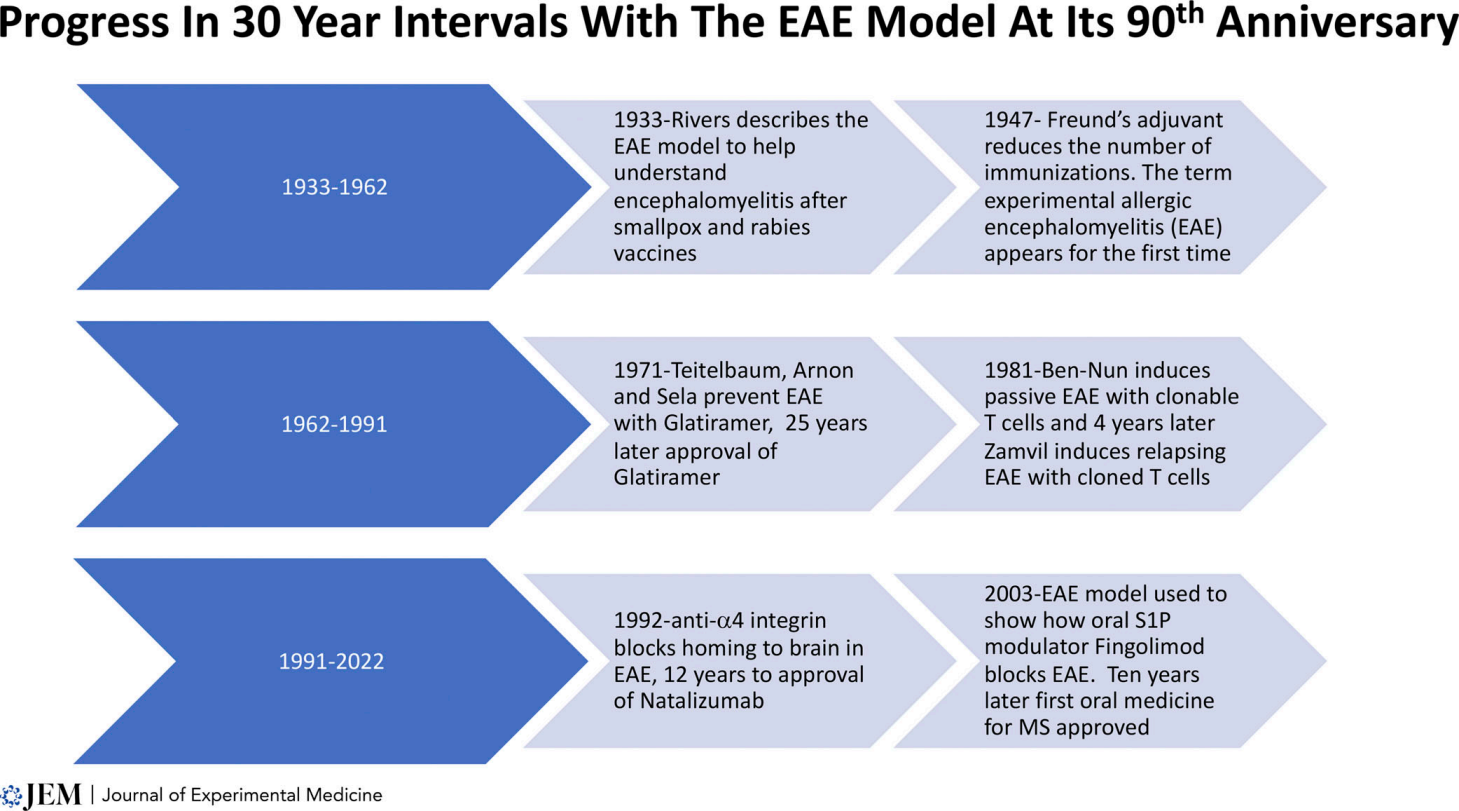
Progress in 30-yr intervals of the EAE model at its 90th anniversary.

We shall see at the centennial of the first publication of the model in *JEM*, a mere decade from now in 2033, what further advances will emanate from this versatile model, now in its 90th year (Fig. 3). And as we think of Thomas Rivers’ efforts, published in this journal 90 yr ago, to explain mechanisms bridging viral infection and immunization against viruses and how these events might trigger neuroinflammation, we might think about a reggae song, Jimmy Cliff’s “Many Rivers to Cross” (Library of Congress 1972). There certainly will be “Many Rivers to Cross” in the next decade as we gain a better understanding of how viruses might trigger inflammation in the brain. EAE might continue to provide that “bridge” to new discoveries based on research involving this exceptionally valuable experimental model.

## Online supplemental material

Table S1 shows annotations, amino acid lengths, and accession numbers for the proteins in Fig. 1.

## Supplementary Material

Table S1 shows annotations, amino acid lengths, and accession numbers for the proteins in Fig. 1.
